# Supplementary material for: Physical Activity during Pregnancy: Comparisons between Objective Measures and Self-Reports in Relation to Blood Glucose Levels
Source: Int J Environ Res Public Health. 2022 Jun 30;19(13):8064. doi: 10.3390/ijerph19138064 (PMC9266138; doi:10.3390/ijerph19138064)
Supplement: Supplementary file 1 [file ijerph-19-08064-s001.zip › ijerph-1718329-supplementary.pdf]

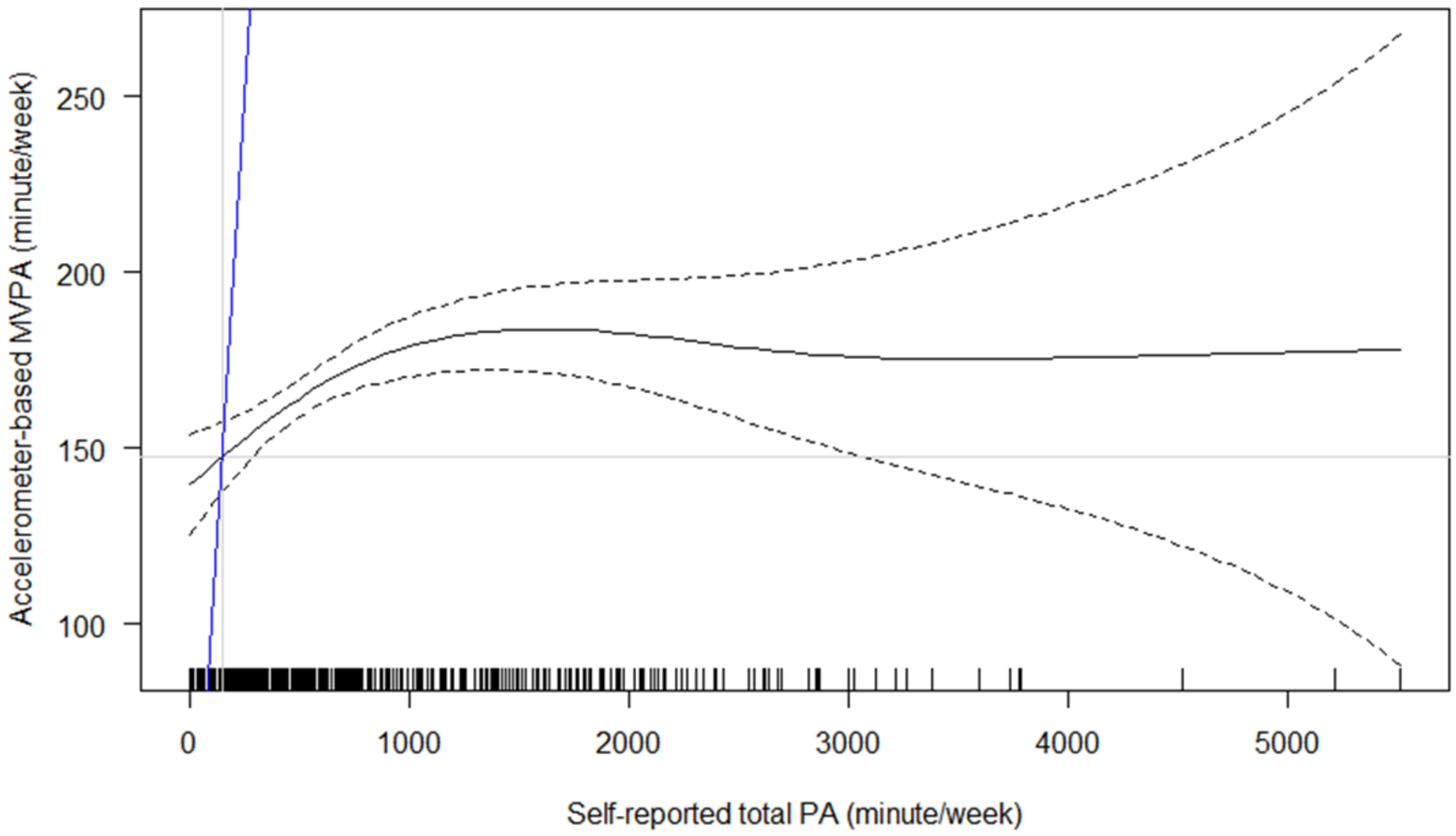

Supplementary Figure S1 Association between self-reported total physical activity (PA) and accelerometer-based moderate-to-vigorous PA (MVPA). *Notes:* The black solid line represents points estimates (and the black dashed lines their 95% confidence intervals). The blue line is a reference line to indicate the hypothesised absolute agreement between two measures.

Supplementary Table S1. Covariate-adjusted (multivariable) associations of sociodemographic and health-related characteristics with accelerometer-based physical activity variables in 2nd trimester (*N* =197)

	**Light PA**			**MVPA**			**Total PA**		
**Variable (unit)**	e^b	**95% CI**	***p***	e^b	**95% CI**	***p***	e^b	**95% CI**	***p***
Gestational age (week)	0.987	(0.952, 1.023)	.478	0.907	(0.832, 0.990)*	.029	0.980	(0.947, 1.013)	.228
Age (year)	0.986	(0.972, 1.001)	.060	1.002	(0.965, 1.041)	.918	0.989	(0.976, 1.002)	.090
Educational attainment									
Lower	Reference			Reference			Reference		
Higher	0.981	(0.853, 1.128)	.787	1.534	(1.098, 2.144)*	.012	1.004	(0.883, 1.142)	.947
Household monthly income									
Lower	Reference			Reference			Reference		
Higher	0.946	(0.875, 1.023)	.162	0.919	(0.759, 1.114)	.391	0.947	(0.882, 1.018)	.141
Employment status									
Full-time	0.995	(0.893, 1.108)	.922	1.593	(1.231, 2.060)***	< .001	1.034	(0.936, 1.143)	.508
Other	Reference			Reference			Reference		
Primipara									
Non-first	Reference			Reference			Reference		
First	0.929	(0.845, 1.022)	.132	1.001	(0.780, 1.284)	.994	0.934	(0.855, 1.020)	.128
In vitro fertilisation									
No	Reference			Reference			Reference		
Yes	1.016	(0.901, 1.145)	.797	0.626	(0.465, 0.843)**	.002	0.980	(0.878, 1.095)	.726
Miscarriage history									
No	Reference			Reference			Reference		
Yes	1.037	(0.939, 1.145)	.469	0.992	(0.773, 1.273)	.948	1.028	(0.938, 1.127)	.553
Pre-pregnancy BMI (kg/m²)	1.012	(0.999, 1.026)	.074	1.021	(0.987, 1.055)	.226	1.013	(1.001, 1.025)*	.040
Normal	Reference#			Reference#			Reference#		
Underweight	0.883	(0.803, 0.972)*	.011	0.905	(0.712, 1.150)	.413	0.883	(0.808, 0.964)**	.006
Overweight/Obese	0.987	(0.856, 1.139)	.861	1.056	(0.743, 1.500)	.763	0.995	(0.872, 1.135)	.941

Notes: All estimates are obtained from generalised linear models with gamma variance and logarithmic function, adjusted for wear time and other variables listed.
PA = physical activity; MVPA = moderate-to-vigorous physical activity; e^b = antilogarithm of regression coefficient, interpreted as the proportional increase (if >1) or decrease (if <1) in outcome variable associated with a 1-unit increase in predictor (for continuous variables) or compared with the reference group (for categorical variables); CI = confidence interval; *p* = p value; BMI = body mass index.
# When this categorised variable was included in the model, the estimates of other variables slightly differ. Nonetheless, the estimates reported in this table are those with the original (uncategorised) BMI.
* *p*< .050; ***p*< .010; ****p*< .001

Supplementary Table S2. Covariate-adjusted (multivariable) associations of socio-demographic and pregnancy-related characteristics with self-reported physical activity variables in 2nd trimester (*N* =197)

				**Non-walking MPA (ii)**											
	**Walking (i)**			**Any vs None (*N*=197)**			**Non-zero (*n*=134)**			**Total MPA (i)**			**Total MVPA (i)**		
**Variable (unit)**	e^b	**95% CI**	***p***	**a*OR***	**95% CI**	***p***	e^b	**95% CI**	***p***	e^b	**95% CI**	***p***	e^b	**95% CI**	***p***
Gestational age (week)	0.952	(0.830, 1.091)	.479	0.904	(0.668, 1.222)	.511	0.944	(0.791, 1.127)	.523	0.941	(0.833, 1.062)	.328	0.944	(0.835, 1.067)	.353
Age (year)	0.965	(0.913, 1.020)	.211	0.964	(0.852, 1.090)	.557	0.984	(0.917, 1.056)	.658	0.970	(0.923, 1.020)	.236	0.970	(0.922, 1.021)	.242
Educational attainment															
Lower	Reference			Reference			Reference			Reference			Reference		
Higher	0.548	(0.319, 0.941)*	.029	2.144	(0.684, 6.719)	.191	0.522	(0.261, 1.042)	.065	0.554	(0.343, 0.896)*	.016	0.547	(0.338, 0.885)*	.014
Household monthly income															
Lower	Reference			Reference			Reference			Reference			Reference		
Higher	0.713	(0.529, 0.961)*	.026	0.590	(0.305, 1.142)	.117	0.934	(0.648, 1.346)	.714	0.734	(0.565, 0.955)*	.021	0.720	(0.553, 0.937)*	.014
Employment status															
Full-time	1.546	(1.020, 2.342)*	.040	0.492	(0.193, 1.254)	.137	0.470	(0.278, 0.783)**	.004	1.047	(0.721, .520)	.810	1.033	(0.711, 1.500)	.866
Other	Reference			Reference			Reference			Reference			Reference		
Primipara															
Non-first	Reference			Reference			Reference			Reference			Reference		
First	0.910	(0.629, 1.317)	.618	1.136	(0.504, 2.560)	.758	1.095	(0.694, 1.730)	.696	0.950	(0.684, 1.318)	.758	0.928	(0.668, 1.290)	.658
In vitro fertilisation															
No	Reference			Reference			Reference			Reference			Reference		
Yes	1.139	(0.716, 1.812)	.581	0.945	(0.342, 2.610)	.913	0.693	(0.391, 1.229)	.210	1.005	(0.667, 1.514)	.980	0.981	(0.651, 1.478)	.925
Miscarriage history															
No	Reference			Reference			Reference			Reference			Reference		
Yes	1.410	(0.974, 2.040)	.069	0.602	(0.271, 1.336)	.212	1.379	(0.825, 2.304)	.220	1.297	(0.932, 1.805)	.124	1.281	(0.920, 1.786)	.143
Pre-pregnancy BMI (kg/m²)	1.005	(0.952, 1.059)	.868	1.214	(1.062, 1.388)**	.005	1.001	(0.940, 1.067)	.965	1.019	(0.971, 1.069)	.449	1.020	(0.973, 1.070)	.408
Normal	Reference#			Reference#			Reference#			Reference#			Reference#		
Underweight	0.699	(0.483, 1.012)	.058	0.323	(0.149, 0.698)**	.004	1.094	(0.667, 1.799)	.722	0.674	(0.487, 0.933)*	.017	0.678	(0.490, 0.940)*	.020
Overweight/Obese	0.861	(0.505, 1.467)	.581	1.824	(0.462, 7.197)	.391	1.293	(0.665, 2.514)	.450	0.957	(0.593, 1.544)	.856	0.964	(0.596, 1.560)	.882

Notes: MPA = moderate physical activity, MVPA = moderate-to-vigorous physical activity; e^b = antilogarithm of regression coefficient, interpreted as the proportional increase (if >1) or decrease (if <1) in outcome variable associated with a 1-unit increase in predictor (for continuous variables) or compared with the reference group (for categorical variables); CI = confidence interval; *p* = p value; BMI = body mass index; a*OR* = adjusted odds ratio.
(i) Estimates are obtained from negative binomial model, adjusted for other variables listed.
(ii) Estimates are obtained from zero-inflated negative binominal regression, adjusted for other variables listed.
# When this categorised variable was included in the model, the estimates of other variables slightly differ. Nonetheless, the estimates reported in this table are those with the original (uncategorised) BMI.
* *p*< .050, ***p*< .010

Supplementary Table S3. Full estimates of Models 2 and 3 in Table 5 of covariate-adjusted associations between accelerometer-based physical activity (PA) variables and 2nd trimester fasting plasma glucose (FPG) (*N* =196)

Outcome variable: 2nd trimester FPG

	**Light PA**						**MVPA**						**Total PA**					
	**Model 2**			**Model 3**			**Model 2**			**Model 3**			**Model 2**			**Model 3**		
**Predictor variable (unit)**	**e*b***	**95% CI**	***p***	***b***	**95% CI**	***p***	**e*b***	**95% CI**	***p***	***b***	**95% CI**	***p***	**e*b***	**95% CI**	***p***	***b***	**95% CI**	***p***
1st trimester FPG (mmol/L)	-	-	-	0.106	(-0.006, 0.219)	.064	-	-	-	0.128	(0.016, 0.240)*	.025	-	-	-	0.108	(-0.003, 0.220)	.057
				**e*b***	**95% CI**	***p***				**e*b***	**95% CI**	***p***				**e*b***	**95% CI**	***p***
PA variable (10min/week)	1.001	(1.00003, 1.002)*	.044	1.001	(0.9998, 1.002)	.089	1.001	(0.997, 1.006)	.484	1.002	(0.999, 1.006)	.347	1.001	(1.0001, 1.002)*	.032	1.001	(0.99997, 1.002)	.058
Age (year)	1.003	(0.986, 1.020)	.770	1.002	(0.986, 1.020)	.771	0.9997	(0.983, 1.017)	.968	1.00003	(0.983, 1.017)	.997	1.002	(0.986, 1.019)	.778	1.003	(0.986, 1.019)	.769
Gestational age (week)	1.019	(0.977, 1.062)	.388	1.016	(0.974, 1.059)	.458	1.019	(0.977, 1.064)	.378	1.017	(0.975, 1.061)	.433	1.021	(0.978, 1.064)	.345	1.018	(0.976, 1.061)	.415
Educational attainment																		
Lower, up to secondary school [n=18]	Reference			Reference			Reference			Reference			Reference			Reference		
Higher, degree and above [n=178]	0.992	(0.844, 1.166)	.920	0.990	(0.843, 1.162)	.902	0.985	(0.836, 1.160)	.854	0.981	(0.834, 1.154)	.818	0.988	(0.840, 1.161)	.879	0.986	(0.840, 1.158)	.865
Household monthly income																		
Lower, less than 10,000 CNY [n=101]	Reference			Reference			Reference			Reference			Reference			Reference		
Higher, 10,000 CNY and above [n=95]	1.068	(0.975, 1.171)	.156	1.054	(0.962, 1.159)	.258	1.058	(0.965, 1.159)	.231	1.044	(0.952, 1.143)	.361	1.070	(0.976, 1.172)	.148	1.056	(0.963, 1.157)	.246
Employment status																		
Full-time [n=158]	0.985	(0.868, 1.116)	.808	0.973	(0.858, 1.103)	.664	0.975	(0.857, 1.109)	.701	0.958	(0.843, 1.090)	.515	0.977	(0.862, 1.108)	.722	0.966	(0.853, 1.095)	.591
Other [n=38]	Reference			Reference			Reference			Reference			Reference			Reference		
Primipara																		
Non-first [n=58]	Reference			Reference			Reference			Reference			Reference			Reference		
First [n=138]	1.055	(0.943, 1.179)	.352	1.052	(0.941, 1.175)	.374	1.041	(0.930, 1.164)	.486	1.040	(0.931, 1.162)	.492	1.055	(0.943, 1.179)	.349	1.052	(0.942, 1.176)	.366
In vitro fertilisation																		
No [n=167]	Reference			Reference			Reference			Reference			Reference			Reference		
Yes [n=29]	0.981	(0.854, 1.127)	.788	0.983	(0.857, 1.128)	.810	0.992	(0.861, 1.143)	.911	0.996	(0.866, 1.146)	.960	0.987	(0.860, 1.134)	.856	0.989	(0.862, 1.134)	.870
Miscarriage history																		
No [n=158]	Reference			Reference			Reference			Reference			Reference			Reference		
Yes [n=38]	1.015	(0.905, 1.138)	.798	1.014	(0.905, 1.136)	.813	1.023	(0.912, 1.149)	.694	1.021	(0.911, 1.144)	.725	1.015	(0.906, 1.138)	.793	1.014	(0.905, 1.136)	.813
Preterm BMI (kg/m²)	1.029	(1.014, 1.045)***	< .001	1.027	(1.011, 1.043)**	.001	1.031	(1.016, 1.047)***	< .001	1.028	(1.012, 1.044)***	<0.001	1.029	(1.013, 1.045)***	<0.001	1.026	(1.011, 1.042)**	.001
Normal [n=138]	Reference#			Reference#			Reference#			Reference#			Reference#			Reference#		
Underweight [n=42]	0.889	(0.792, 0.997)*	.045	0.902	(0.803, 1.012)	.079	0.871	(0.776, 0.977)*	.018	0.871	(0.776, 0.977)*	.018	0.892	(0.794, 1.001)	.052	0.906	(0.807, 1.017)	.093
Overweight/Obese [n=16]	1.181	(0.999, 1.396)	.052	1.167	(0.988, 1.379)	.069	1.175	(0.992, 1.392)	.061	1.175	(0.992, 1.392)	.061	1.180	(0.998, 1.394)	.053	1.166	(0.988, 1.377)	.070

Notes: b = regression coefficient; e*b* = antilogarithm of regression coefficient, interpreted as the proportional increase (if >1) or decrease (if <1) in outcome variable associated with a 1-unitincrease in independent variable; CI = confidence interval; *p* = p value; CNY = Chinese yuan; BMI = body mass index.
Model 2: Estimates are obtained from general linear models adjusted for other variables listed.
Model 3: Model 2 plus 1st trimester FPG
'-' indicates not applicable.
# When this categorised variable was included in the model, the estimates of other variables slightly differ. Nonetheless, the estimates reported in this table are those with the original (uncategorised) BMI.
* *p*< .050, ***p*< .010, ****p*<.001

Supplementary Table S4. Covariate-adjusted association between 1st trimester fasting plasma glucose (FPG) and accelerometer-based physical activity (PA) in 2nd trimester (*N* =197)

	**LPA**			**MVPA**			**Total PA**		
**Variable**	**e*b***	**95% CI**	***p***	**e*b***	**95% CI**	***p***	**e*b***	**95% CI**	***p***
1st trimester FPG (unit: mmol/L)	1.111	(1.013, 1.219)*	.025	0.817	(0.639, 1.046)	.108	1.083	(0.993, 1.181)	.071

Notes: e*b* = antilogarithm of regression coefficient, interpreted as the proportional increase (if >1) or decrease (if <1) in outcome variable associated with a 1-unitincrease in independent variable; a*OR* = adjusted odds ratio; CI = confidence interval; *p* = p value.
* *p*< .050

Supplementary Table S5. Covariate-adjusted association between 1st trimester fasting plasma glucose (FPG) and self-reported physical activity (PA) in 2nd trimester (*N* =197)

				**Non-walking MPA**											
	**Walking**			**Any vs None (*N*=197)**			**Non-zero (*n*=134)**			**Total MPA**			**Total MVPA**		
**Variable**	**e*b***	**95% CI**	***p***	**aOR**	**95% CI**	***p***	**e*b***	**95% CI**	***p***	**e*b***	**95% CI**	***p***	**e*b***	**95% CI**	***p***
1st trimester FPG (unit: mmol/L)	1.414	(1.009, 1.982)*	.044	0.976	(0.441, 2.157)	.952	0.872	(0.524, 1.452)	.599	1.337	(0.981, 1.824)	.066	1.296	(0.951, 1.766)	0.101

Notes: a*OR* = adjusted odds ratio; CI = confidence interval; *p* = p value.
* *p*< .050
